# Ultra-High Electromagnetic Absorption Property of One-Dimensional Carbon-Supported Ni/Mo_2_C and Polyvinylidene Fluoride

**DOI:** 10.3389/fchem.2019.00427

**Published:** 2019-06-20

**Authors:** Shan Gao, Jie Feng, Guang-Sheng Wang, Ben-Liang Liang

**Affiliations:** ^1^School of Chemistry, Beihang University, Beijing, China; ^2^College of Materials Science and Engineering, Zhejiang University of Technology, Hangzhou, China

**Keywords:** carbon-supported Ni/Mo_2_C (Ni/Mo_2_C-C) nanocomposite, high absorbing properties, hot-molding technique, the synergistic effect, promising EM wave absorber

## Abstract

A novel one-dimensional carbon-supported Ni/Mo_2_C (Ni/Mo_2_C-C) nanocomposite with excellent electromagnetic wave absorption properties was successfully synthesized by annealing NiMoO_4_@PDA directly, and then the (Ni/Mo_2_C-C)/polyvinylidene fluoride (PVDF) composites were fabricated using a simple blending and hot-molding technique. An excellent reflection loss (RL) of −55.91 dB at 9.28 GHz with a low filler loading (15 wt%) and effective bandwidth (RL < −10 dB) of 14.12 GHz in the thickness range of 1.5–5.0 mm was obtained. Dielectric loss is considered to be the dominant mechanism of (Ni/Mo_2_C-C)/PVDF, which was confirmed by the Debye relaxation process and attenuation theory.

**Graphical Abstract:**
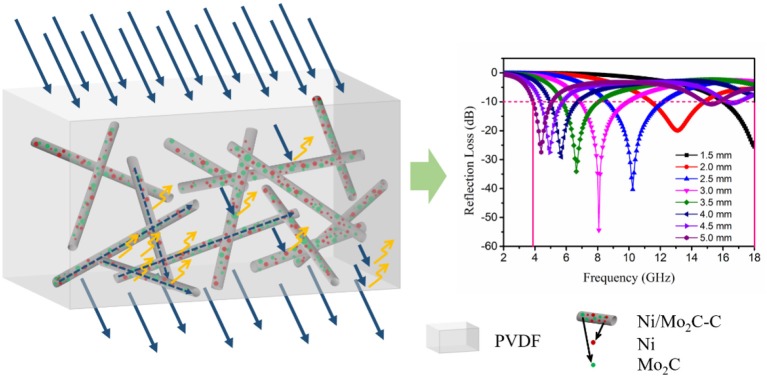
Description of electromagnetic wave absorption performance of Ni/Mo2C-C nanocomposite.

## Introduction

In recent years, electronic equipment has been widely used in military, civil, aerospace and other fields as a medium of information dissemination. However, electromagnetic waves were generated during the reception and transmission of electronic devices, causing problems such as electromagnetic (EM) interference, electromagnetic radiation, electromagnetic leakage and so on, which represent multiple hazards to the security of the country's political, economic and military stability and to human health. Loss-based microwave absorption (MA) materials have been identified among the most effective solutions (Liu et al., 2012, 2016b; Xia et al., 2013; Zhao et al., 2016; Xu et al., 2017). According to the loss mechanism of microwave energy, MA could be divided into two kinds: dielectric- and magnetic-loss. Thus, an increasing number of studies are focused on improving the electromagnetic wave absorption properties of materials by adjusting the dielectric and/or magnetic loss. (Ren et al., 2013; Jiang et al., 2014; Liang et al., 2014; Li et al., 2017).

The transition metal nickel has been widely studied as a magnetic loss MA material due to its high magnetic permeability in the GHz frequency band, ease of preparation, and low cost (Yang et al., 2013; Wen et al., 2014; Han et al., 2015; Zhao et al., 2015b). Zhao et al. (2015a) synthesized the magnetic-dielectric core-shell heterostructure composites with the core of Ni submicron spheres and the shell of SnO_2_ nanorods (Ni/SnO_2_). The results show that the paraffin- Ni/SnO_2_ composites exhibit strong microwave absorbing intensities (−45.0 dB at 13.9 GHz) with a filler loading of 50 wt%. Shi et al. (2016) synthesized a core-shell structure of Ni@BaTiO_3_ nanocomposite and found that the optimal reflection loss (RL) of Ni@BaTiO_3_-parrafin composites is −42.3 dB at 10.6 GHz with a filler loading of 60 wt%. It can be seen from these result that the electromagnetic wave absorbing material prepared from magnetic loss material as a single component has a large filling rate in application, and does not satisfy the requirements of lightweight and portability. However, the dielectric absorbing materials compensate for these defects due to their easy polarization and excellent electrical conductivity (Wang and Dang, 2005; Wang et al., 2010; Cheng et al., 2011; Qiang et al., 2016). In addition, the cooperative effect on the dissipation of electromagnetic wave energy between magnetic loss and dielectric loss and the interfacial polarization are good for achieving excellent electromagnetic wave absorption performance (Liu et al., 2013). Molybdenum carbide (Mo_2_C) is an important member of early transition metal carbide, and is recognized for its excellent conductivity and chemical stability, becoming a new star in the microwave absorption field. Dai and his co-workers (Dai et al., 2018) have confirmed that Mo_2_C is an excellent dielectric-loss electromagnetic wave absorption material with Multiple polarization mechanisms. The minimum RL value of a porous-carbon-based Mo_2_C/C nanocomposite can reach −49.19 dB at matching thickness of 2.6 mm. Therefore, we believe Mo_2_C is a potential material as a dielectric-loss component in nanocomposite absorption materials.

The morphology of nanomaterials is also an important factor affecting the wave absorption performance (Ma et al., 2018; Yu et al., 2018). In recent years, many studies have confirmed that the one-dimensional (1D) morphology of materials has a great influence on the improvement of absorption properties (Deng and Han, 2007; Liu et al., 2016a; Xu et al., 2018). 1D nanomaterials have large length-diameter ratio, high thermal stability and good mechanical properties, which can especially transmit electromagnetic wave directionally (Wu et al., 2016a). Kuang et al. (2016) found that a three-dimensional (3D) conductive path for the dissipative current can be formed by the connection of 1D structures disorderly dispersed in a matrix, which directly leads to a significant increase in conduction loss.

In summary, the dielectric-magnetic matching components and the one-dimensional structure greatly contribute to the improvement of absorbing properties. In our study, we first synthesized uniform nickel molybdate (NiMoO_4_) nanowires with one-dimensional structure by hydrothermal method. Then, the surface of NiMoO_4_ was coated with a controlled thickness of dopamine hydrochloride by a simple stirring-soaking method. A one-dimensional precursor with a core-shell structure was synthesized. After that, carbon-supported Ni/Mo_2_C (Ni/Mo_2_C-C) nano-composites with dielectric-magnetic loss medium were prepared by annealing of the precursor in argon atmosphere, which retained the 1D structure by the supporting of carbon and is a combination of dielectric and magnetic components. This allowed for the completion of the dual design based on the structure and composition.

## Materials and Methods

### Preparation of Porous Ni_x_S_y_

#### Materials

All chemicals used in this process are commercially available without further purification. Nickel chloride hexahydrate (NiCl_2_·H_2_O), trishydroxymethylaminomethane (Tris), and dopamine hydrochloride (C_8_H_12_ClNO_2_) were supplied by Xilong Chemical Reagent Co. Ltd. (Guangdong, China). Ammonium molybdate ((NH_4_)_6_Mo_7_O_24_), hydrochloric acid (HCl), absolute ethanol (C_2_H_5_OH), polyethylene glycol (PEG-400), ether (C_4_H_10_O), polyvinylidene fluoride (PVDF), and N, N-dimethylformyl (DMF) were supplied by Beijing Chemical Factory (Beijing, China). Ammonia was supplied by Tianjin Jinke Fine Chemical Research Institute (Tianjin, China).

#### Synthesis of 1D NiMoO_4_ Nanowires

1D NiMoO_4_ nanowires was synthesized by simple hydrothermal method. 0.14 mmol of (NH_4_)_6_Mo_7_O_24_ and 1 mmol of NiCl_2_·H_2_O were dissolved in 13 mL of deionized water, respectively. The NiCl_2_·H_2_O solution was added to the (NH_4_)_6_Mo_7_O_24_ solution and stirred for 10 min. The pH of the mixed solution was adjusted to 7 using ammonia water and hydrochloric acid. 2 mL of PEG-400 was added under magnetic stirring. The clear solution was transferred into a 50 mL Teflon-lined stainless autoclave and kept at 140°C for 12 h. After reaction, the solution was centrifuged, washed, and dried to obtain NiMoO_4_ nanowires.

#### Preparation of Tris-HCl Buffer Solution (pH = 8.5)

0.1 mol TRIS was dissolved in 500 mL of deionized water. And then, 2.45 mL HCl was added. The mixed solution was fixed to a 2 L volumetric flask.

### Synthesis of NiMoO_4_@PDA Nanorods

The NiMoO_4_ nanorods (80 mg) were dispersed into 200 mL Tris-HCl buffer aqueous solution and then 60, 80, 100, and 120 mg of dopamine hydrochloride were added under magnetic stirring, respectively. The reaction was stirred continuously at room temperature for 4.5 h and the NiMoO4@PDA nanocomposites were obtained by centrifugation and washed with deionized water. Different ratios of NiMoO_4_@PDA were named NiMoO_4_@PDA-60, NiMoO_4_@PDA-80, NiMoO_4_@PDA-100 and NiMoO_4_@PDA-120.

### Synthesis of Carbon-Supported Ni/Mo_2_C Nanocomposites

Synthesized NiMoO_4_@PDA nanorods were treated at 800°C for 2 h with a heating rate of 2°C min^−1^ under Ar atmosphere to get the final carbon-supported Ni/Mo_2_C (Ni/Mo_2_C-C) nanocomposites. For comparison, the catalysts with 60, 80, 100, and 120 mg dopamine hydrochloride added were also prepared under other conditions unchanged.

### Characterization

X-ray diffraction (XRD) characterization was carried out on XRD-6000 (Shimadzu, Japan). The morphology and microstructures were analyzed by scanning electron microscope (SEM, Questar Quanta 250 FEG), transmission electron microscope (TEM, JEOL JEM-2100F) and field emission scanning electron microscope (FESEM, JEOL JSM-7500F). Raman spectroscopy (Horiba Jobin Yvon, LabRAM HR800) was used to record the properties of samples in the range of 400 ~ 2000 cm^−1^ with an excitation wavelength of 514.5 nm. X-ray photoelectron spectroscopy (XPS) was collected on an ESCALab MKII X-ray photoelectron spectrometer. The thermal stability was analyzed by thermogravimetric analysis (TG, STA 449F3, Netzsch, Germany) and the test temperature is from room temperature to 800 degrees Celsius with a heating rate of 10°C/min in air flow. The specimen for microwave measurement was prepared by uniformly blending the samples with PVDF in different mass fractions and then pressing them into a ring-like compact structure (with a 3.04 mm inner diameter, 7.00 mm outer diameter). The relative complex permeability and permittivity of samples + PVDF composites were measured by a vector network analyzer (Agilent, PNA 5244A).

## Results and Disscussion

**Figure 1:**
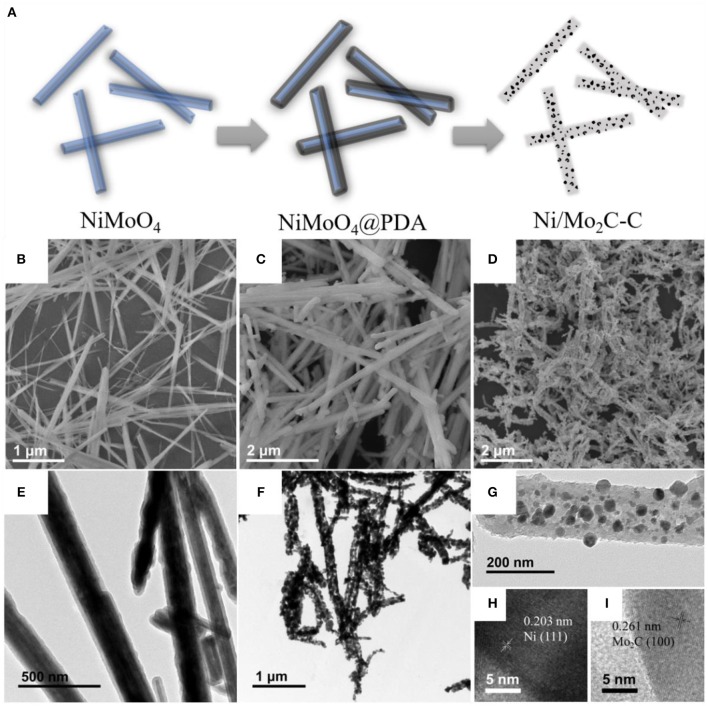
**(A)** Illustration of the synthesis of Ni/Mo_2_C-C from NiMoO_4_ nanowires, SEM images of **(B)** NiMoO_4_ nanowires, **(C)** NiMoO_4_@PDA and **(D)** Ni/MoO_4_, TEM images of **(E)** NiMoO_4_@PDA and **(F,G)** Ni/Mo_2_C-C and **(H,I)** HRTEM images of Ni/Mo_2_C-C in part.

**Figure 2:**
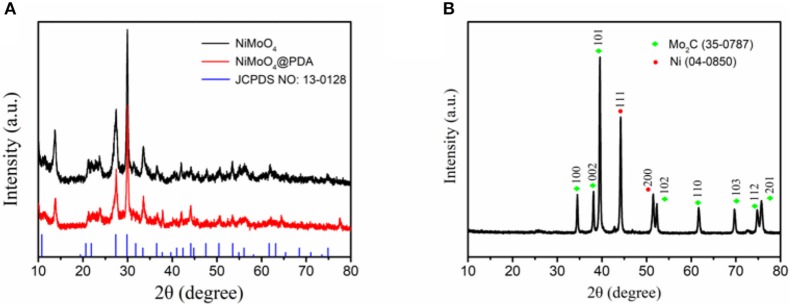
XRD patterns of **(A)** NiMoO_4_ and NiMoO_4_@PDA, and **(B)** Ni/Mo_2_C-C.

An illustration of the synthesis of the Ni/Mo_2_C-C nanocomposites from NiMoO_4_ nanowires is shown in Figure 1A, and corresponding SEM images are shown in Figures 1B–D, respectively. NiMoO_4_ one-dimensional nanowires with a length about 4 μm were first synthesized by simple hydrothermal process and their XRD pattern was basically consistent with the NiMoO_4_·H_2_O (JCPDS no. 13-0128) (as shown in Figure 2A). Then a uniform polydopamine (PDA) shell was coated on the NiMoO_4_ nanowires to prepare NiMoO_4_@PDA (as shown in Figure 1E). The XRD pattern indicates that the crystal phase of NiMoO_4_ was not changed. The addition of dopamine hydrochloride had a great influence on the PDA shell and the uniform coating facilitated the uniformity of the annealed product. The additional amount of dopamine hydrochloride was changed to adjust the uniformity of coating. We found that the NiMoO_4_@PDA-120 own a more uniform shell compared to NiMoO_4_@PDA-60, NiMoO_4_@PDA-80 and NiMoO_4_@PDA-100, and the thickness was about 23 nm (as shown in Figure S1). The obtained NiMoO_4_@PDA-120 powder was transferred into a tube furnace and annealed at 800 °C for 2 h under an Ar atmosphere, which eventually led to the generation of the 1D Ni/Mo_2_C-C nanocomposites (Yu et al., 2017). SEM and TEM images showed that the obtained 1D Ni/Mo_2_C-C composite has a rod-like structure that the particulate Ni and Mo_2_C encapsulated within the carbon shells (in Figures 1D,F,G). A clear SEM image shows defects present on the surface of the annealed product (in Figure S2), which was beneficial for the absorption of electromagnetic waves (Fang et al., 2016; Han et al., 2017). XRD pattern of as-obtained Ni/Mo_2_C-C is shown in Figure 2B, which indicates the presence of Ni phase (JCPDS no. 04-0850) and Mo_2_C phase (JCPDS no. 35-0787). The spacings of 0.203 nm and 0.261 nm in the HRTEM image also correspond to the (111) plane of Ni and the (100) plane of Mo_2_C, respectively (in Figures 1H,I) (Mckone et al., 2013). The Raman spectrum shows two distinct peaks located at about 1350 and 1590 cm^−1^, which can be assigned to the D and G bands of carbon, respectively (in Figure S3) (Eklund et al., 1995; Zhang et al., 2010). Moreover, the intensity of the D band was stronger than the G band, demonstrating the presence of significant structural defects within Ni/Mo_2_C-C (Liu et al., 2017). The TG results show that when the temperature was about 700°C, the mass of the product tended to be constant with an increasing of the annealing temperature, indicating a stable product formation (in Figure S4), which led to 800°C being considered a suitable annealing temperature.

**Figure 3:**
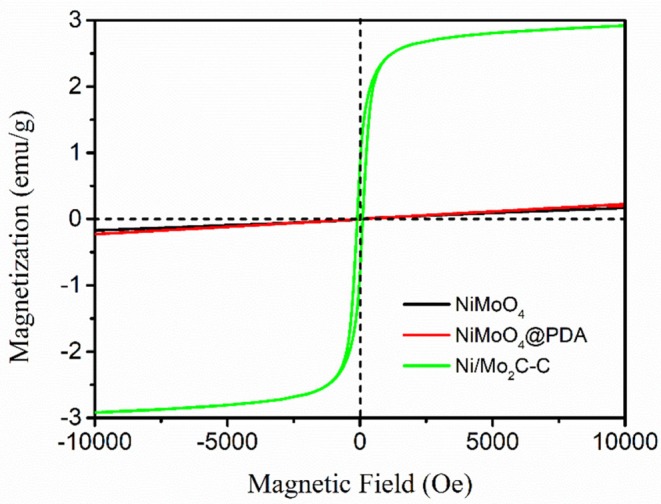
Room-temperature hysteresis loops of NiMoO_4_, NiMoO_4_@PDA and Ni/Mo_2_C-C.

The magnetic properties of the as-synthesized NiMoO_4_, NiMoO_4_@PDA and Ni/Mo_2_C-C nanoparticles were measured at room temperature. As observed in Figure 3, both NiMoO_4_ and NiMoO_4_@PDA samples show weak paramagnetism, but Ni/Mo_2_C-C sample shows a typical hysteresis loop in its magnetic behavior. The saturation magnetization (Ms), remanent magnetization (Mr), and coercivity (Hc) values were 2.9 emu/g, 0.9 emu/g and 100.2 Oe, respectively. The differences in magnetic properties between NiMoO_4_, NiMoO_4_@PDA and Ni/Mo_2_C-C are mainly due to the formation of Ni. Compared with the magnetic properties of elemental nickel reported in other literatures (Li et al., 2010; Liu et al., 2016a), the Ms value of Ni/Mo_2_C-C in this paper is significantly reduced. The result is usually attributed to the support structure of carbon with low crystallinity and the small content of Ni in the nanocomposite.

**Figure 4:**
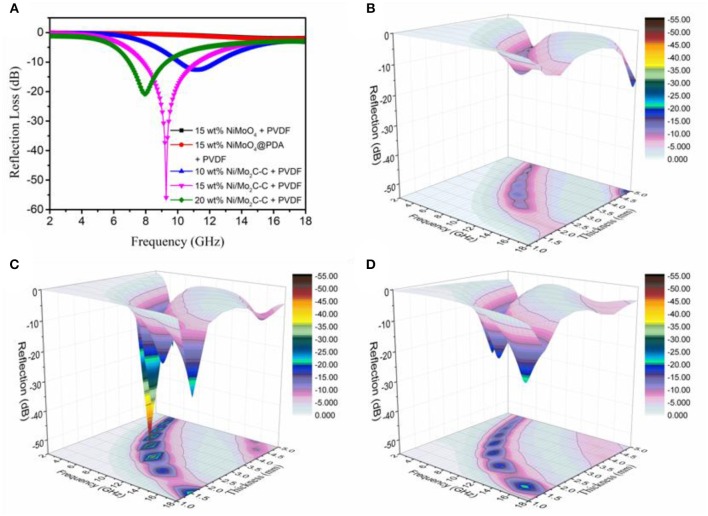
**(A)** Microwave RL curves of different composites with 2.7 mm thicknesses. Three-dimensional presentations of the RLs of (Ni/Mo_2_C-C)/PVDF composites at various thicknesses in 2–18 GHz frequency range with loadings of Ni/Mo_2_C-C **(B)** 10 wt%, **(C)** 15 wt%, and **(D)** 20 wt%.

**Figure 5:**
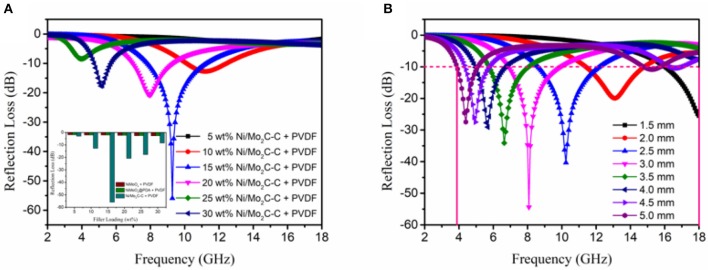
**(A)** Reflection loss of (Ni/Mo_2_C-C)/PVDF composite absorbers at various loadings of Ni/Mo_2_C-C, and reflection loss of different samples at various filler loadings (insert), **(B)** The microwave RL curves of (Ni/Mo_2_C-C)/PVDF composites with a filler loading of 15 wt% at different thicknesses in the frequency range of 2–8 GHz.

To study the microwave absorption property, the reflection loss (RL) values of the as-obtained samples were calculated from the measured relative complex permittivity (ε_*r*_ = $\varepsilon_{r}^{\prime }$ − *j*$\varepsilon_{r}^{\prime \prime }$) and complex permeability (μ_*r*_ = $\mu_{r}^{\prime }$ − *j*$\mu_{r}^{\prime \prime }$) values under the normal incidence of the electromagnetic field. According to the transmission line theory, the RL can be defined with the following equations (Che et al., 2004; Wu et al., 2016b):

(1) $$\begin{array}{l} R=20\log\left|\frac{Z_{in}-1}{Z_{in}+1}\right| \end{array}$$

where Z_in_ is the input characteristic impedance, which can be expressed as:

(2) $$\begin{array}{l} Z_{in}=\sqrt{\frac{\mu_{r}}{\varepsilon_{r}}}\tan h\left[j\left(\frac{2f\pi d}{c}\right)\sqrt{\mu_{r}\varepsilon_{r}}\right] \end{array}$$

where ε_*r*_ and μ_*r*_ are the complex permittivity and permeability of the composite absorber, respectively, *f* is the frequency, *d* is the thickness of the absorber, and *c* is the velocity of light in free space. Thus, Figure 4A shows the calculated RL of different composite absorbers with different filler loadings at 2.5 mm thickness in the 2–18 GHz range, which were obtained from equation (1) and (2). It is clear that the RL of the (Ni/Mo_2_C-C)/PVDF composite is much stronger than those of other composites under the filler loading of 15 wt%, and this regularly also applies when the filler loadings are 5, 10, 20, 25 and 30 wt% (shown in the insert of Figure 5A). (Ni/Mo_2_C-C)/PVDF composites with 15 wt% Ni/Mo_2_C-C especially present the most enhanced EM wave absorption property. The minimum reflection loss value can reach−55.91 dB at 9.28 GHz, and the effective frequency bandwidth (RL < −10 dB) is 3.46 GHz from 7.81 to 11.27 GHz. Furthermore, when the loadings of Ni/Mo_2_C-C nanorods increase, the maximum RL values of (Ni/Mo_2_C-C)/PVDF composites initially increase and then decrease with a shifting of frequency to a lower-frequency region (in Figure 5A). This indicates that the frequency of microwave attenuation can be tuned by controlling the loading of Ni/Mo_2_C-C nanorods. In addition, Figure 5B shows that the (Ni/Mo_2_C-C)/PVDF composites attained a reflection loss of < −10 dB in the frequency range from 3.8 to 18.0 GHz for thickness in the range of 3.8–5.0 mm, which confirms that this kind of composite can act a potential candidate in the field of broadband microwave absorption. The electromagnetic wave absorption performances of various carbon-based materials are compared in Table 1. It can be seen that Ni/Mo_2_C-C nanorod is an ideal electromagnetic wave absorbing material with low filling amount, high reflection loss, small thickness and effective absorption bandwidth.

**Table 1:** The electromagnetic wave absorption performance of various carbon-based materials.

**Samples**	**Filler loading (wt%)**	**RL (dB)**	**Thickness (mm)**	**< −10 dB (GHz)**	**References**
FeCo@C	50	−16.8	3.0	-	Tan et al., 2019
Fe_3_O_4_@C@MoS_2_	20	−51.6	2.0	6.2	Zhang et al., 2019b
MnO_2_@CNFs	20	−42.8	4.5	2.8	Zhang et al., 2019a
MWCNTs/CeO_2_	50	−51.1	2.6	3.4	Wu et al., 2019
Co-C/MWCNTs	25	−50.0	1.8	2.4	Shu et al., 2019
CoNC/CNT	15	−44.3	4.7	1.7	Xu et al., 2019
Fe_3_O_4_/CNT	50	−42.9	2.0	2.8	Li et al., 2019
Fe_3_O_4_/C	50	−54.6	4.3	2.8	Wu et al., 2018
Fe/Fe3O4@C	50	−29.3	3.9	-	Feng et al., 2018
Ni/Mo_2_C-C	15	−55.9	2.7	3.5	This work

The three-dimensional presentations of the RL (Figures 4B–D) show the calculated theoretical RL of the (Ni/Mo_2_C-C)/PVDF composites with different thicknesses (2–5 mm) in the range of 2–18 GHz with the loadings of 10 wt%, 15 wt%, and 20 wt%, respectively. This indicates that the microwave absorption ability can be tuned effectively by controlling the thickness and filler contents of the composite absorbers.

**Figure 6:**
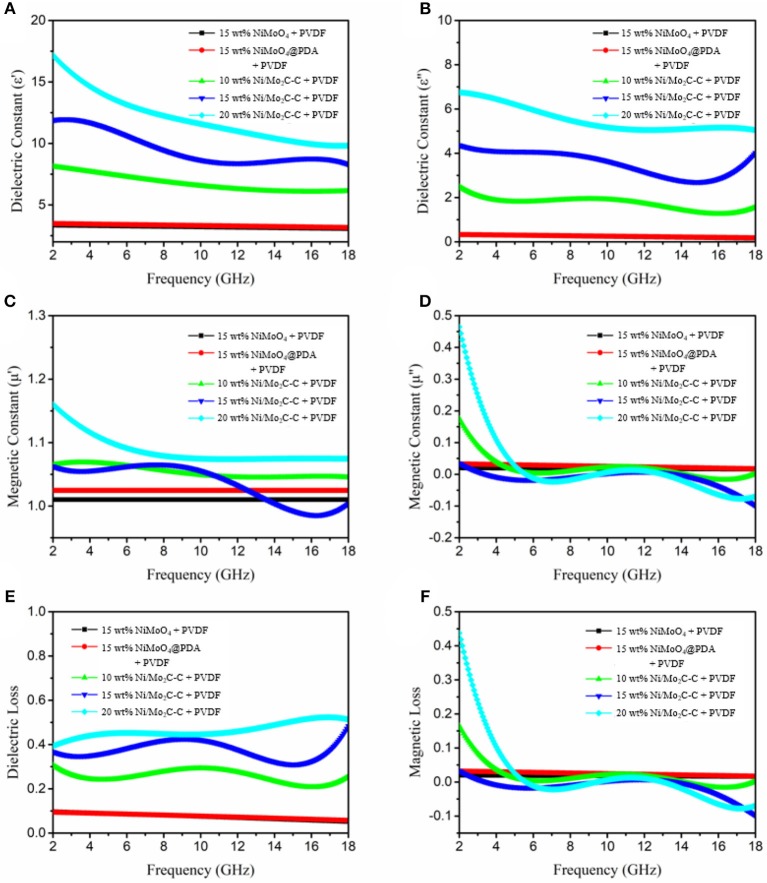
Frequency dependence of **(A)** real parts and **(B)** imaginary parts of the complex permittivity, **(C)** real parts and **(D)** imaginary parts of the complex permeability, **(E)** dielectric loss, and **(F)** magnetic loss.

To investigate the possible mechanisms and effects to the enhancement of microwave absorption, Figures 6A–D show the frequency dependence of electromagnetic parameters including complex permittivity real part (ε′), permittivity imaginary part (ε″), permeability real part (μ′), and permeability imaginary part (μ″) of all samples (Jazirehpour and Ebrahimi, 2015; Qiu and Qiu, 2015). It can be intuitively seen that the real parts and imaginary parts of the complex permittivity of Ni/Mo_2_C-C nanorods are larger than NiMoO_4_ and NiMoO_4_@PDA when the filler loading is the same, and show increasing trends with the increasing ratio of Ni/Mo_2_C-C nanorods. The dielectric properties of NiMoO_4_ and NiMoO_4_@PDA may be due to their poor conductivity. The real parts and imaginary parts of the magnetic permeability of NiMoO_4_ and NiMoO_4_@PDA have remained mostly constant in the measured frequency range, but some fluctuations appear on the Ni/Mo_2_C-C nanorods. This result may be caused by the magnetic hysteresis and the relaxation of magnetic moments procession of Ni/Mo_2_C-C nanorods, as mentioned above.

In addition, the dielectric loss tangents (tan δ_ε_ = ε″/ε′) and magnetic loss tangents (tan δ_μ_ = μ″/μ′) of all samples were calculated as shown in Figures 6E,F. The higher tan δ_ε_ values illustrate that the dielectric loss is the main influencing factor for absorption of electromagnetic wave performance. However, Debye dipolar relaxation is the main reason for dielectric loss of inorganic–organic composites absorb microwaves. The relationship between complex permittivity can be deduced as Guo et al. (2012):

(3) $$\begin{array}{l} {\left(\varepsilon^{\prime }-\frac{\varepsilon_{s}+\varepsilon_{\infty }}{2}\right)}^{2}+{\left(\varepsilon^{\prime \prime }\right)}^{2}={\left(\frac{\varepsilon_{s}-\varepsilon_{\infty }}{2}\right)}^{2} \end{array}$$

where ε_*s*_ and ε_∞_ are the static permittivity and relative dielectric permittivity at the high-frequency limit, respectively. Thus, the plot of ε′ vs. ε″ is a single semicircle called Cole-Cole semicircle, of which each represents a relaxation process (Wen et al., 2013). In Figure S6, there are three Cole—Cole semicircles in the ε′ - ε″ curves for PVDF with 10 wt% Ni/Mo_2_C-C, representing the contribution of the Debye relaxation processes of PVDF and Ni/Mo_2_C-C (in Figure S6a). When the loading reached 15 wt%, four Cole–Cole semicircles were found, indicating that, with the increase of filler loadings, the appearance of interfacial polarization between Ni, Mo_2_C, C, and PVDF, and the formation of Ni/Mo_2_C-C conductive network (in Figure S6b). As the filler loading continued to increase, Ni/Mo_2_C-C and PVDF gradually became a whole conductor, which was caused by an initial reduction of the Cole–Cole semicircle and two semicircles were found with a filler loading of 25 wt%, which is due to pure PVDF and the electric loss, respectively. For the analysis of the Debye dipolar relaxation, it is shown that the proper concentration of the filler loading is beneficial to the absorbing properties of the composite, which is derived from the induced relaxation process of the nano-absorber, the interfacial polarization between the nano-absorber and PVDF, and the polarization of the conductive network formed by the nanorods absorber. Attenuation constant is considered to be another important factor influencing dielectric loss, which can be written according to transmission line theory as Hong et al. (2015):

(4) $$\alpha =\frac{\sqrt{2}\pi f}{c}\times \sqrt{(\mu^{\prime \prime }\varepsilon^{\prime \prime }-\mu^{\prime }\varepsilon^{\prime })+\sqrt{{\left(\mu^{\prime \prime }\varepsilon^{\prime \prime }-\mu^{\prime }\varepsilon^{\prime }\right)}^{2}+{\left(\mu^{\prime }\varepsilon^{\prime }-\mu^{\prime \prime }\varepsilon^{\prime }\right)}^{2}}}$$

where *f* is the frequency of the electromagnetic wave and *c* is the velocity of light. As shown in Figure S5, larger attenuation constants result in stronger dielectric losses, which is beneficial for electromagnetic wave absorption performance.

Being different from the significant effect of dielectric loss, weak magnetic loss was also investigated. In this study, the main role of the magnetic loss is considered to be natural resonance, which can be expressed by the following equation (Lv et al., 2015):

(5) $$\begin{array}{l} H_{a}=\frac{4|K_{1}|}{3\mu_{0}M_{s}} \end{array}$$

where *H*_*a*_ is the anisotropy energy, |*K*_1_| is the anisotropic coefficient, and Ms is the saturation magnetization. As shown in Figure 3, Ni/Mo_2_C-C nanorods with hysteresis have a certain saturation magnetization, which results in the generation of natural resonance (Afghahi and Shokuhfar, 2014; Wen et al., 2014; Wang et al., 2017).

## Conclusions

In conclusion, we have successfully synthesized a one-dimensional carbon-supported Ni/Mo_2_C (Ni/Mo_2_C-C) nanocomposite by annealing NiMoO_4_@PDA, which was pre-prepared by coating the PDA on the nanowire-like NiMoO_4_ surface prepared using a simple hydrothermal method. A series of (Ni/Mo_2_C-C)/PVDF composites were prepared with different filler loadings of Ni/Mo_2_C-C using a simple blending and hot-molding technique. The maximum reflection loss of 15 wt% (Ni/Mo_2_C-C)/PVDF composites was found to reach as high as −55.91 dB at 9.28 GHz with a matching thickness of 2.7 mm, and the mechanism of enhancement was explained. The Cole–Cole semicircles suggested that the interface and the synergistic effect between Ni/Mo_2_C-C and PVDF promote high absorbing properties. Furthermore, the weak magnetic loss caused by natural resonance is also considered to contribute to the improvement of absorbing performance. The (Ni/Mo_2_C-C)/PVDF composite, with its advantages of strong absorption, small thickness, broadband absorption and adjustability, can be considered a promising EM wave absorber.

## Data Availability

The raw data supporting the conclusions of this manuscript will be made available by the authors, without undue reservation, to any qualified researcher.

## Author Contributions

SG performed the main experimental operation and drafted the manuscript. JF performed the data analyses. G-SW contributed to the conception of the study and financial support. B-LL helped to revised the manuscript and approved the final version.

### Conflict of Interest Statement

The authors declare that the research was conducted in the absence of any commercial or financial relationships that could be construed as a potential conflict of interest.
