# Supplementary material for: Ultra-High Electromagnetic Absorption Property of One-Dimensional Carbon-Supported Ni/Mo2C and Polyvinylidene Fluoride
Source: Front Chem. 2019 Jun 20;7:427. doi: 10.3389/fchem.2019.00427 (PMC6595158; doi:10.3389/fchem.2019.00427)
Supplement: Supplementary file 1 [file Data_Sheet_1.docx]

**Supplementary Materials**

**Ultra-high Electromagnetic Absorption Property of One-dimensional Carbon-supported Ni/Mo_2_C and Polyvinylidene Fluoride**


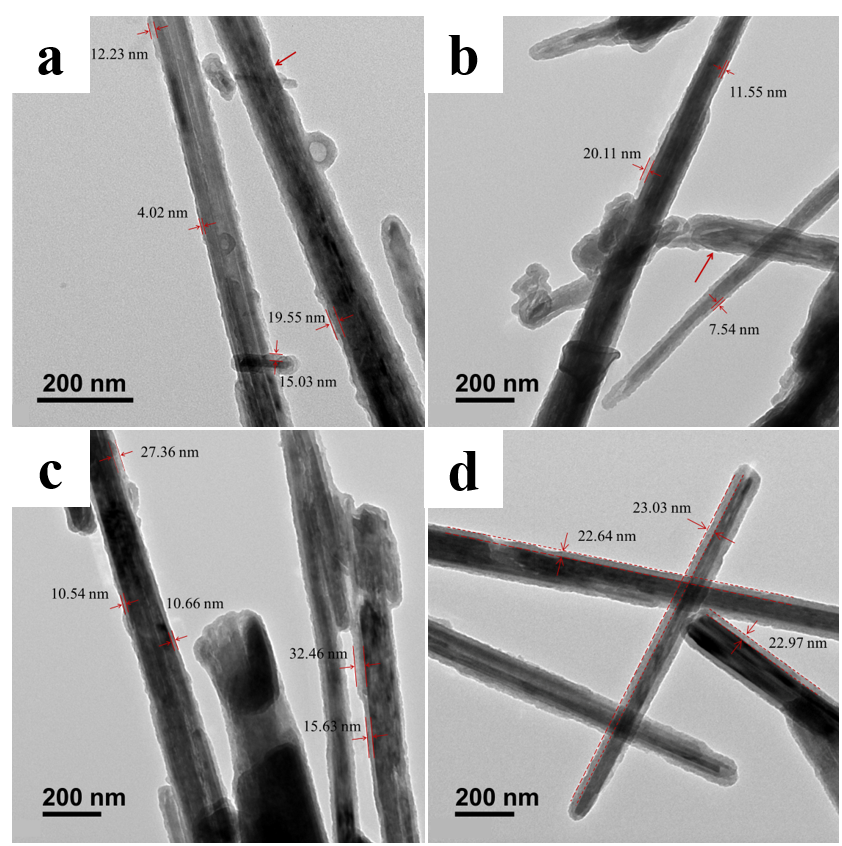


**Figure S1 | The SEM images of (a) NiMoO_4_@PDA-60, (b) NiMoO_4_@PDA-80, (c) NiMoO_4_@PDA-100 and (d) NiMoO_4_@PDA-120.**


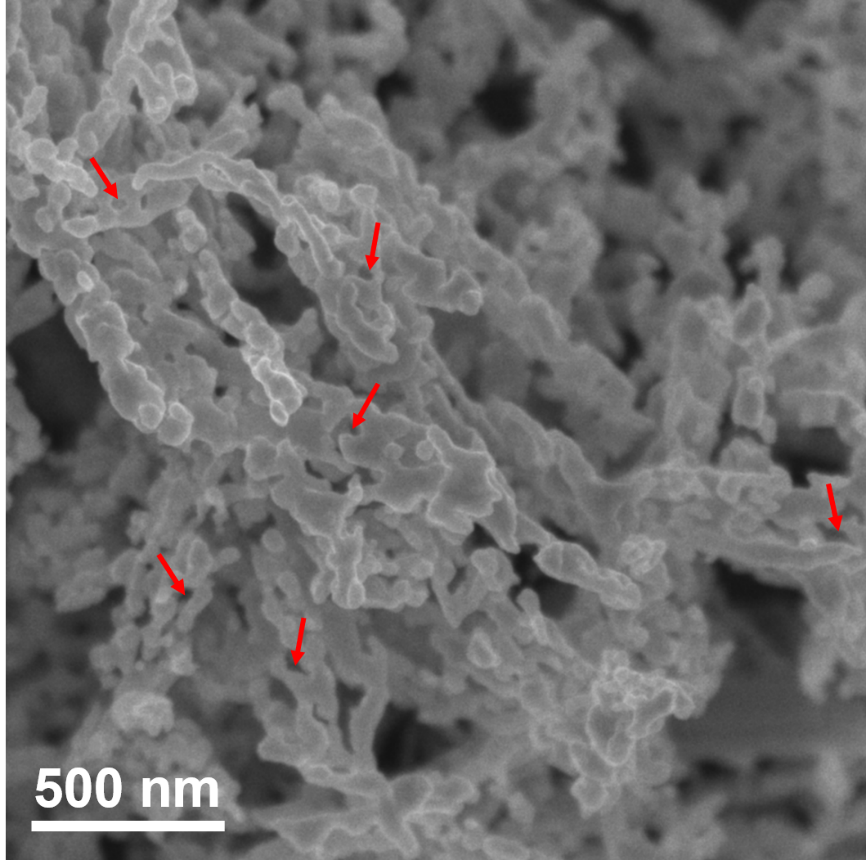


**Figure S2 | SEM image of Ni/Mo_2_C-C.**


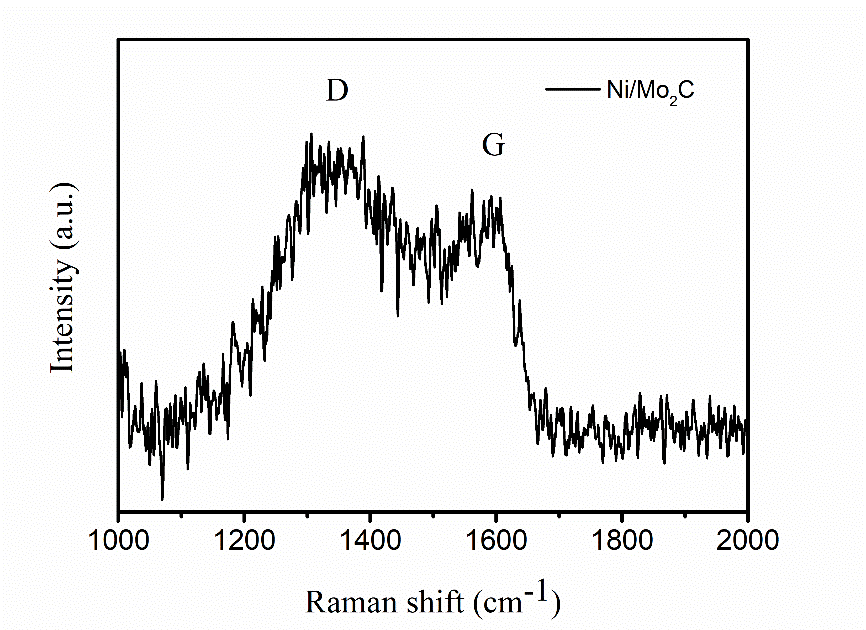


**Figure S3 | Raman spectrum of Ni/Mo2C-C.**


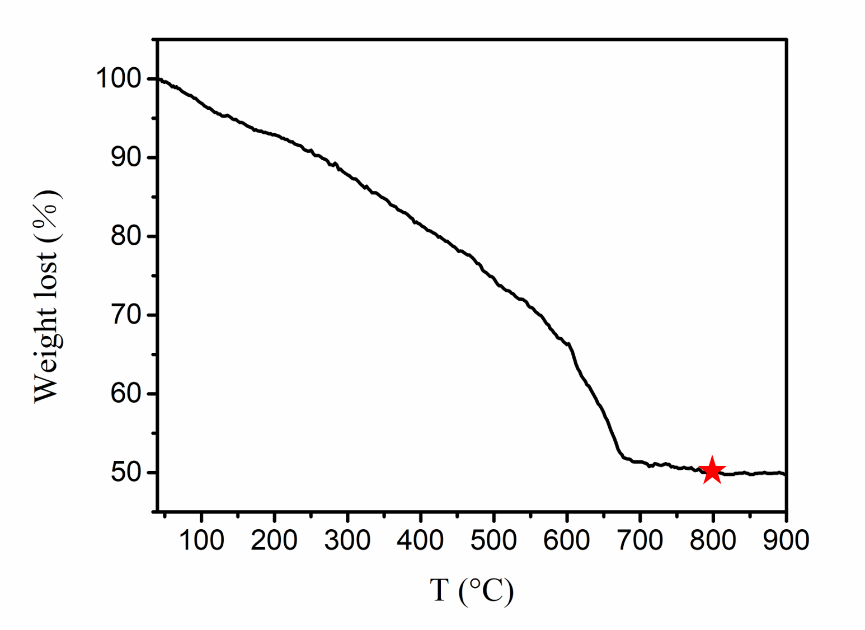


**Figure S4 | TG curve of NiMoO_4_.**


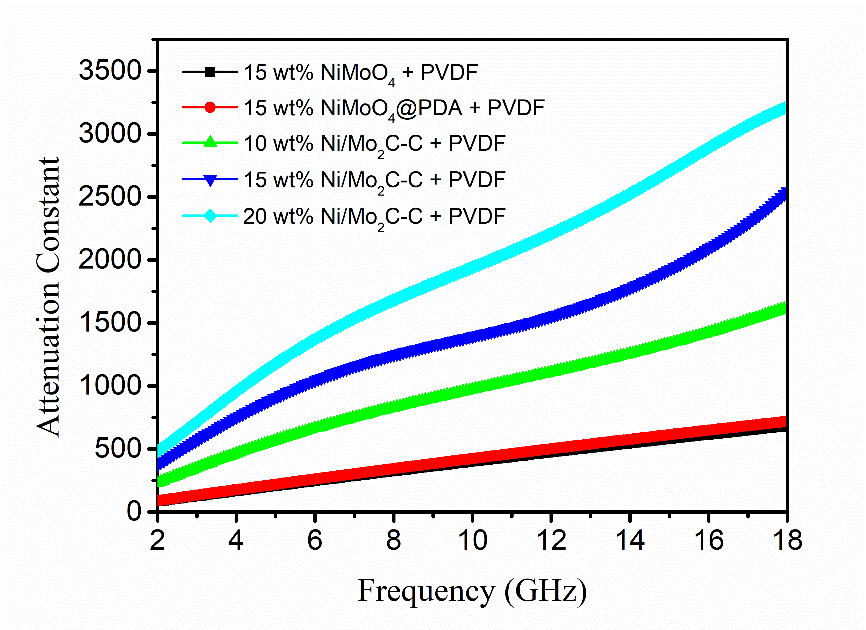


**Figure S5 | Attenuation constants of various samples.**


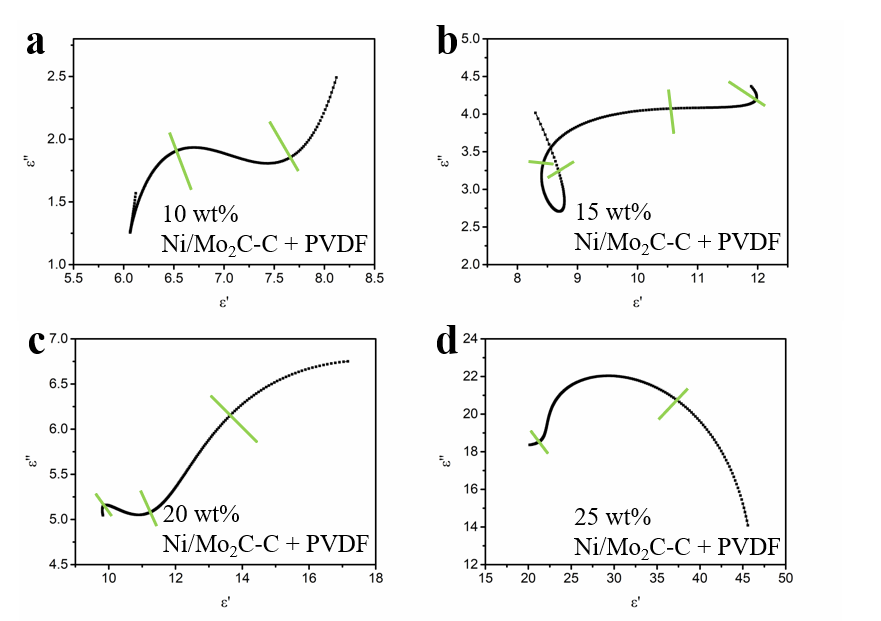


**Figure S6 | The Cole–Cole semicircle of the composites with different filler loadings: (a) 10 wt%, (b) 15 wt%, (c) 20 wt%, and (d) 25 wt%.**
